# Multiple relapses of mantle cell lymphoma as different extranodal lesions: A case report

**DOI:** 10.3892/mi.2025.284

**Published:** 2025-10-31

**Authors:** Masahiro Manabe, Daisuke Aohara, Daiki Mukai, Satoru Nanno, Ki-Ryang Koh

**Affiliations:** 1 Department of Hematology, Osaka General Hospital of West Japan Railway Company, Osaka 545-0053, Japan; 2 Department of Respiratory Medicine, Osaka City General Hospital, Osaka 534-0021, Japan

**Keywords:** mantle cell lymphoma, extranodal disease, ocular adnexa, soft tissue, cardiac involvement

## Abstract

Mantle cell lymphoma (MCL) usually affects the lymph nodes; however, extranodal involvement is also common, particularly in Waldeyer's ring and the gastrointestinal tract, spleen and bone marrow. Other organs that may be affected include the skin, endocrine glands, lungs and central nervous system, with these sites most commonly affected by relapsing disease. However, in rare, extranodal MCL has arisen in the ocular adnexa, soft tissue and heart. The present study reports the case of a 75-year-old male patient, who experienced repeated relapses of MCL, involving ocular adnexa, soft-tissue and cardiac lesions, which are rare. The aim of the present case report was to demonstrate that clinicians should be aware of the various extranodal sites at which MCL can arise, and monitor patients carefully during and after treatment.

## Introduction

Mantle cell lymphoma (MCL) is a mature B-cell neoplasm derived from the mantle zone of lymphoid follicles and is typically composed of small- to medium-sized monomorphic cells. Its diagnostic characteristics include the t(11;14)(q13;q32) translocation and cyclin D1 overexpression, the former causing the latter (1,2). MCL usually involves the lymph nodes; however, extranodal involvement is also common, particularly in Waldeyer's ring and the gastrointestinal tract, spleen and bone marrow. Other organs that may be affected include the skin, endocrine glands, lungs and central nervous system, with these sites most commonly affected by relapsing disease (1). In addition, a wide variety of extranodal lesions, such as submandibular duct and endobronchial lesions, have been reported in recent years (3,4). However, in rare cases, extranodal MCL has arisen in the ocular adnexa, soft tissue and heart. The present study report the case of a patient with MCL, which demonstrated metachronous extranodal recurrence in the conjunctiva, soft tissue and right atrium. The clinical characteristics of the disease at each site, as well as the treatment course are described.

## Case report

**Figure 1:**
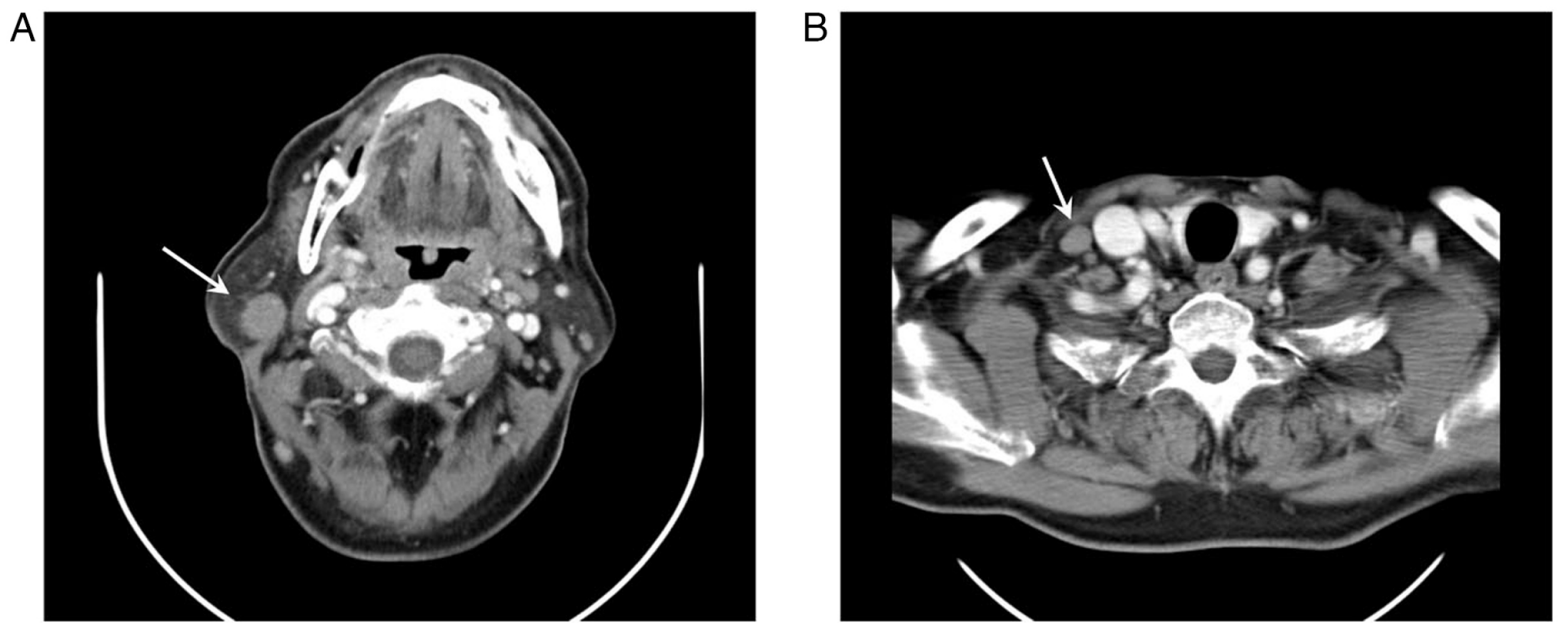
Computed tomography revealed (A) a swollen right cervical lymph node (arrow) and (B) a supraclavicular lymph node (arrow).

**Figure 2:**
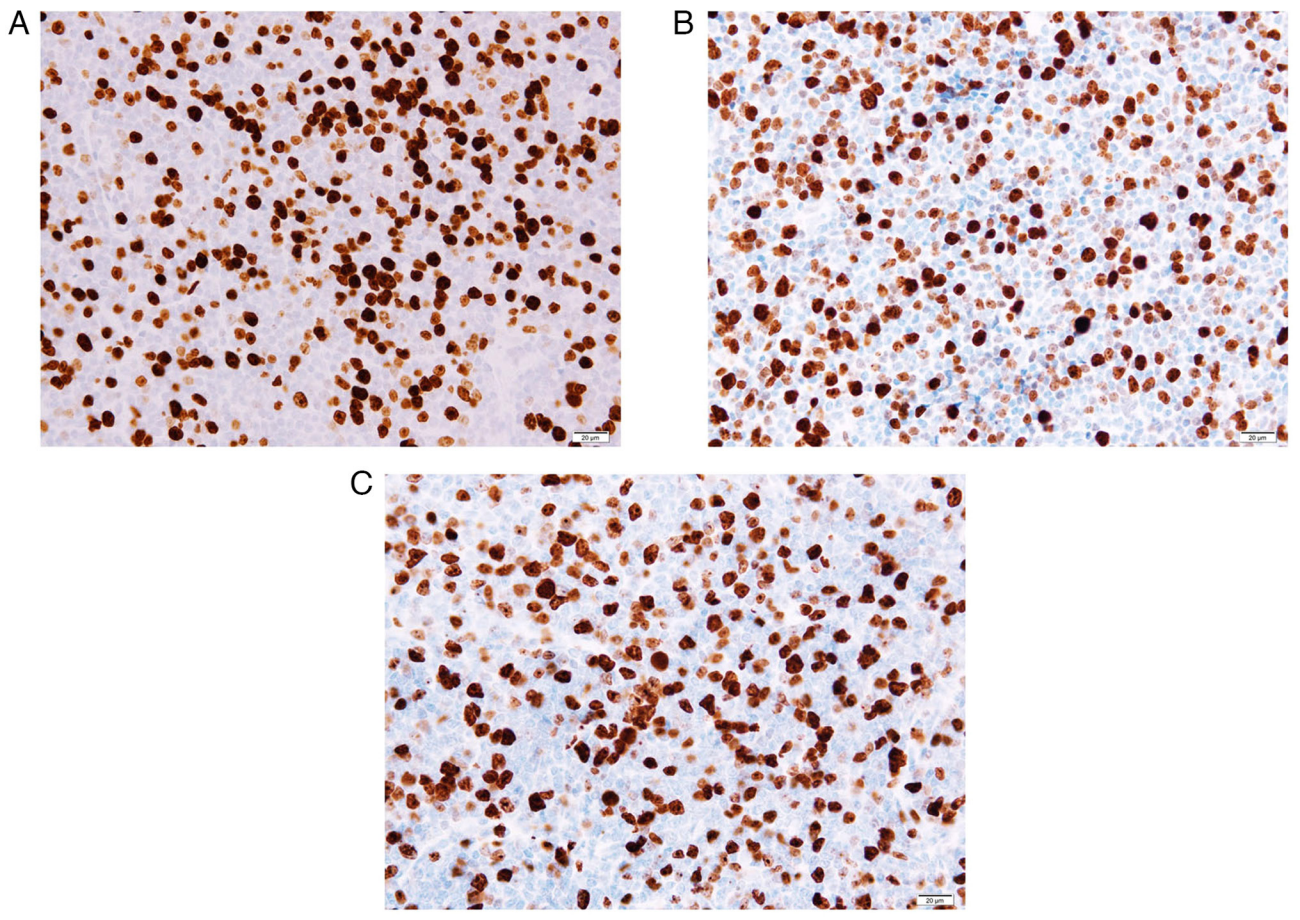
Pathological examinations of the (A) lymph node, (B) eyelid, and (C) popliteal mass revealed positivity for Ki-67 (magnification, x400).

**Figure 3:**
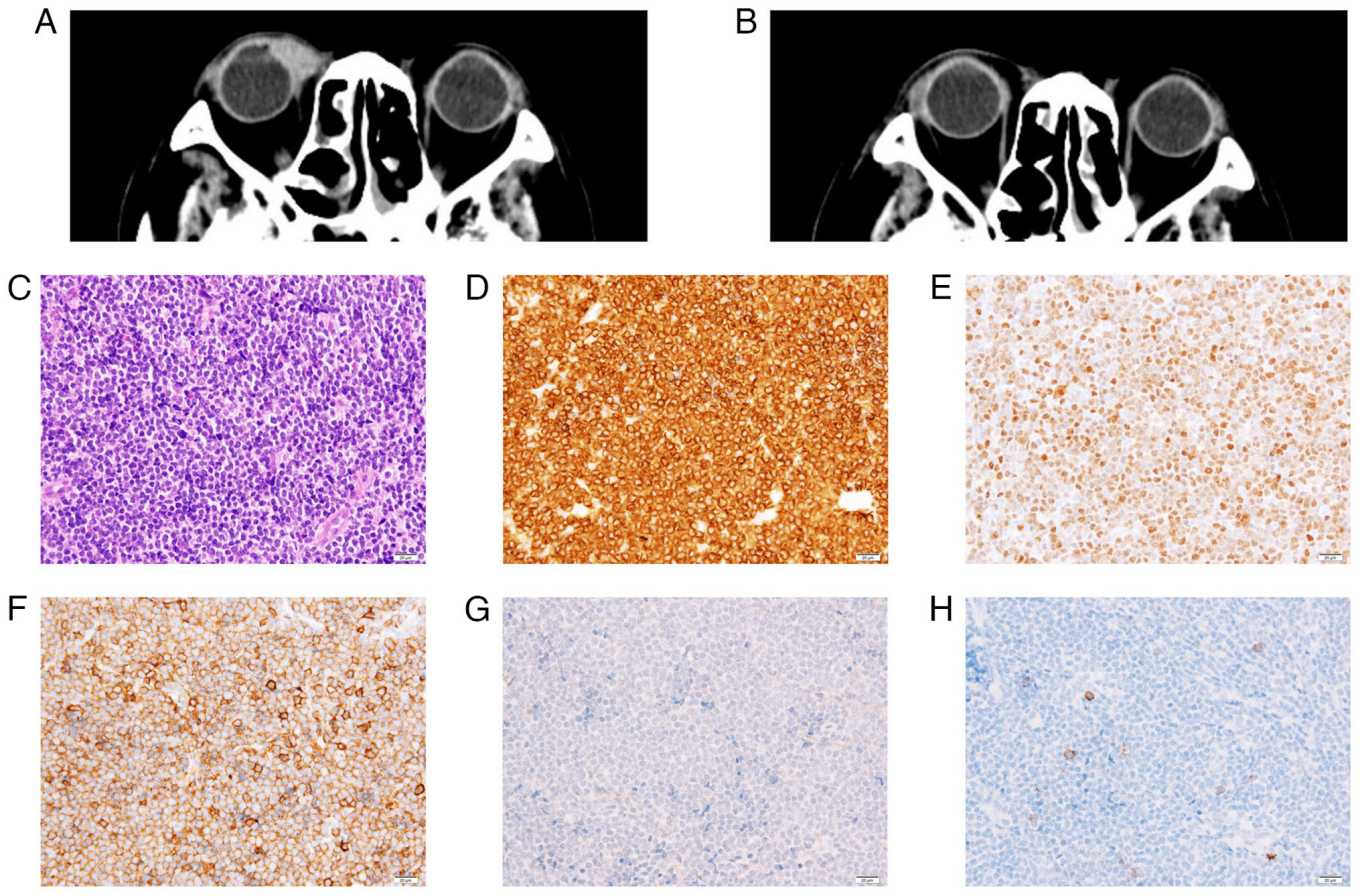
(A) Computed tomography scan illustrating a mass in the right orbit. (B) Following radiotherapy, the mass disappeared. (C) The conjunctival mass was composed of diffuse, dense sheets of atypical cells (hematoxylin and eosin staining; magnification, x400). The abnormal cells were immunopositive for (D) Bcl-2 (magnification, x400), (E) cyclin D1 (magnification, x400), and (F) CD5 (magnification, x400), and negative for (G) CD10 (magnification, x400), and (H) CD23 (magnification, x400).

A 75-year-old male patient with a history of systemic MCL presented with right cervical and supraclavicular lymph node swelling, and was referred to the Osaka General Hospital of West Japan Railway Company in July, 2018. His MCL was first diagnosed 10 years prior (December, 2007), and remission had been achieved with chemotherapy, involving rituximab, cyclophosphamide, vincristine, doxorubicin and dexamethasone. A physical examination did not reveal any other superficial lymph node swelling. The performance status of the patient was categorized as 0 according to the Eastern Cooperative Oncology Group performance status scale. Laboratory examinations revealed a white blood cell count of 4.2x10^9^/l, a hemoglobin concentration of 13.5 g/dl, a platelet count of 209x10^9^/l, a lactate dehydrogenase level of 191 U/l (reference range, 106-211 U/l) and a soluble interleukin-2 receptor level of 405 U/ml (reference range, 145-519 U/ml). On a computed tomography (CT) scan, cervical and supraclavicular lymph node swelling were observed (Fig. 1); however, no disease, including splenomegaly, was found at other sites. A biopsy of the swollen cervical lymph node demonstrated the massive proliferation of monotonous lymphoid cells with irregular nuclear contours. The mass blocks were fixed in a solution containing 10% formaldehyde (Yuaikasei Co., Ltd.) in 0.01 M phosphate-buffered saline (Muto Pure Chemicals Co., Ltd.) for 2 h at room temperature. Following fixation, the tissue blocks were loaded into the Tissue-Tek^®^ TEC 6 (Sakura Finetek Japan Co., Ltd.) and subsequently embedded in paraffin. Formalin-fixed paraffin-embedded sections were cut (3-µm-thick) using a YAMATO REM-710 microtome (Yamato Kohki Industrial Co., Ltd.). The subsequent dewaxing process included sequential treatments with xylene, anhydrous ethanol, a decreasing concentration gradient of ethanol, and water. Following this, the sections were immersed in hematoxylin staining solution (Muto Pure Chemicals Co., Ltd.) for 5 min at room temperature and then differentiated with 0.3% acid alcohol before being incubated with 0.6% ammonia for 1 min at room temperature. Eosin staining solution (Muto Pure Chemicals Co., Ltd.) was applied for 3 min, followed by dehydration with ethanol and xylene. Finally, the samples were mounted with neutral gum to prepare the slides. Immunohistochemical staining revealed that the lymphoid cells were positive for Bcl-2 (cat. no. 413141; Nichirei Biosciences, Inc.), CD5 (cat. no. 413251; Nichirei Biosciences, Inc.), CD79a (cat. no. 413161; Nichirei Biosciences, Inc.), CD20 (cat. no. 760-2531; Roche Diagnostics) and cyclin D1 (cat. no. 413521; Nichirei Biosciences, Inc.) and negative for CD10 (cat. no. 413261; Nichirei Biosciences, Inc.) and CD3 (cat. no. 413591; Nichirei Biosciences, Inc.). Ki-67 (cat. no. 760-4286; Roche Diagnostics) staining stained >20% of the cells with varying intensity (Fig. 2). Fluorescence *in situ* hybridization (FISH) analysis was not performed due to a lack of tissue samples. A pathological diagnosis of relapse of known MCL was made. A bone marrow examination did not demonstrate any evidence of bone marrow invasion. At this time, the MCL international prognostic index (MIPI) score of the patient was calculated as ~5.9954, indicating that he was in the intermediate risk group (5). Since the patient had stage II disease, he was subjected to radiotherapy. Volumetric modulated arc therapy (2 Gy x 20 fractions) resulted in complete remission. At 1 year following radiotherapy (October, 2019), he presented with right conjunctival swelling. No ophthalmological symptoms other than discomfort, such as pain, vision loss, exophthalmos, or epiphora, were observed. A CT scan demonstrated an enlarged right conjunctiva. An excisional biopsy of the conjunctiva was performed, which revealed the diffuse infiltration of monotonous lymphocytes. Phenotypically, they were positive for CD5, Bcl-2, Ki-67 and cyclin D1, but negative for CD10 and CD23 (cat. no. 413611; Nichirei Biosciences, Inc.), and these findings confirmed the diagnosis of relapsed MCL (Figs. 2 and 3). Bone marrow aspiration, a whole-body CT scan and fluorodeoxyglucose-positron emission tomography (FDG-PET) did not identify any other disease. The patient was commenced on external-beam radiotherapy (2 Gy daily up to 40 Gy in 4 weeks), which led to complete remission.

**Figure 4:**
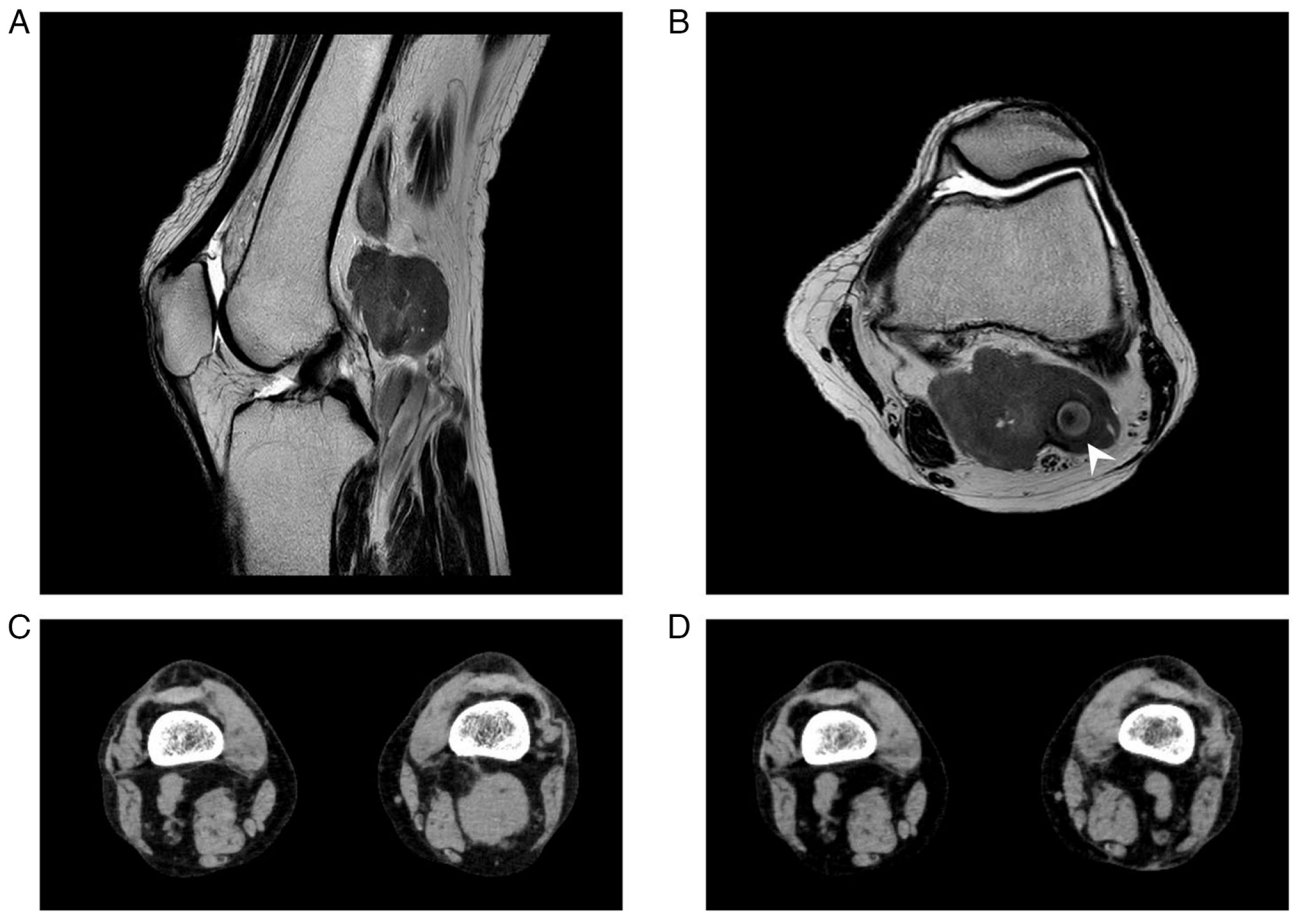
(A and B) Magnetic resonance images (T2-weighted images) of the left popliteal fossa are shown; (A) sagittal view and (B) axial view. (B) The mass exhibited homogenous signal intensity and encased the popliteal artery (arrowhead). A computed tomography scan revealed (C) a left popliteal mass, and (D) the mass regressed following external-beam radiotherapy.

**Figure 5:**
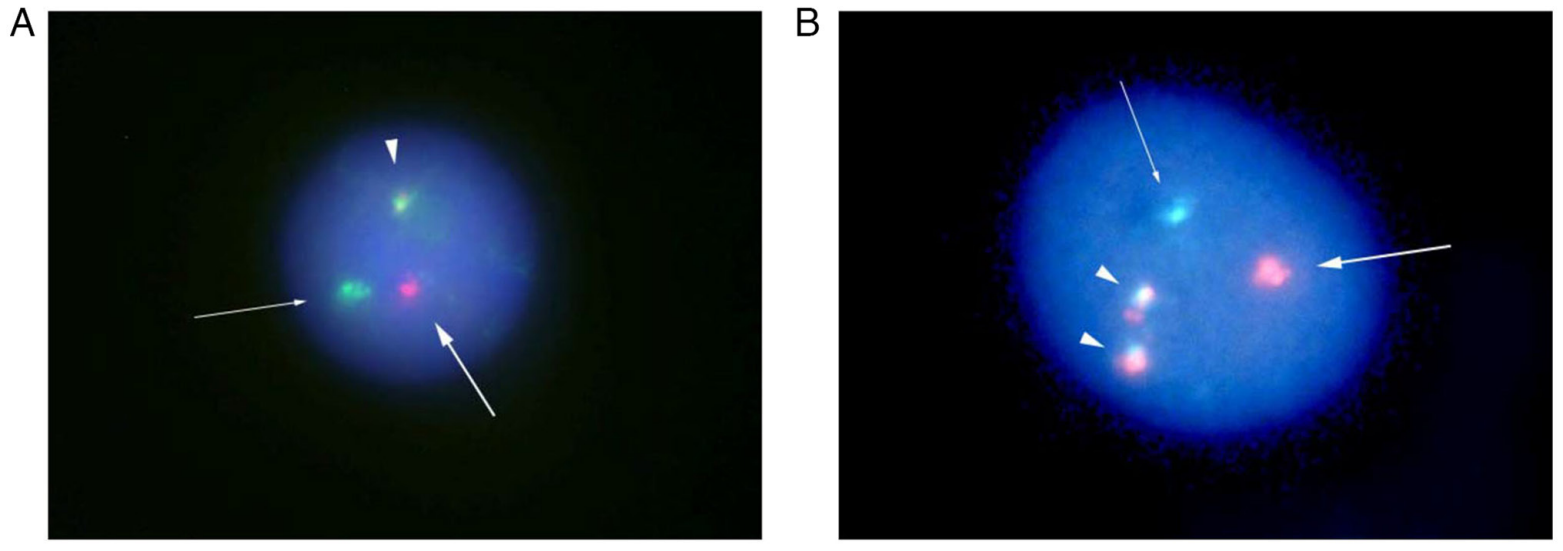
FISH analyses of (A) the popliteal mass and (B) bone marrow cells using CCND1/IGH fusion probe. (A) In 949 interphase nuclei out of 1,000 analyzed cells, 1 CCND1/IGH fusion signal (arrowhead), 1 CCND1 red signal (thick arrow) and 1 IGH green signal (thin arrow) were detected. (B) In 12 interphase nuclei out of 1,000 analyzed cells, two CCND1/IGH fusion signals (arrowheads), one CCND1 red signal (thick arrow), and one IGH green signal (thin arrow) were detected. FISH, fluorescent *in situ* hybridization.

In February, 2022, the patient complained of a painless mass in the left popliteal fossa. Magnetic resonance imaging (MRI) of the left knee revealed a lesion of 54x34 mm in size localized in the popliteal fossa, which exhibited intermediate signal intensity on T1- and T2-weighted images (Fig. 4). A surgical biopsy of the popliteal mass was performed and demonstrated the same pathological findings as the previous examination of the conjunctival mass, and Ki-67 staining produced a positive result (Fig. 2). FISH of interphase nuclei detected CCND1/IGH fusion signals using CCND1/IGH fusion probe (Vysis IGH/CCND1 XT Dual Color, Dual Fusion FISH Probe kit; Abbott Molecular Inc.) in 949 of the 1,000 analyzed cells (Fig. 5). Hence, the patient was diagnosed with recurrent MCL. FDG-PET revealed FDG accumulation in the popliteal mass, and a bone marrow examination revealed no evidence of bone marrow involvement; therefore, the disease was diagnosed as stage I. Since the disease had recurred twice, chemotherapy was suggested as a suitable therapeutic strategy; however, the patient preferred radiotherapy. Thereafter, external-beam radiotherapy (2 Gy x 20 fractions) was performed, resulting in complete remission, which lasted for 2 years.

**Figure 6:**
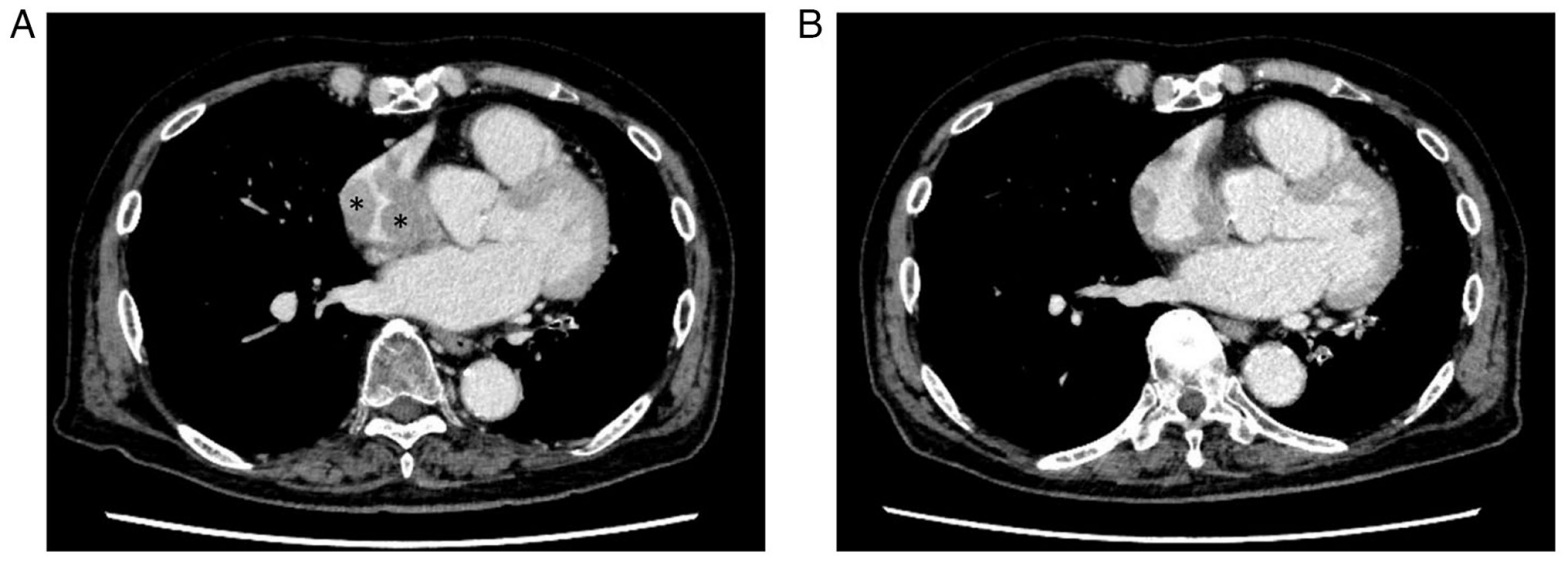
A computed tomography scan revealed (A) mass lesions in the right atrium (asterisks); (B) the disease improved within 4 months with ibrutinib monotherapy.

Although the patient remained well for ~2 years following radiotherapy for the popliteal mass (January, 2025), he subsequently developed swollen preauricular and cervical lymph nodes. A CT scan demonstrated no abnormal findings in the conjunctiva or popliteal fossa; however, a right atrial mass was newly found in addition to the swollen preauricular and cervical lymph nodes (Fig. 6). An FDG-PET scan also demonstrated positivity at the same sites. Although transthoracic echocardiography was performed, the right atrial mass could not be detected. An electrocardiography demonstrated a sinus rhythm with supraventricular premature contraction; i.e., there was no change from previous examinations. FISH of the bone marrow cells of the patient using the CCND1/IGH fusion probe revealed fusion signals in 12 interphase nuclei out of 1,000 analyzed cells (Fig. 5). Therefore, a diagnosis of recurrent MCL was made, and the disease was staged as stage IV. Since the bone marrow examination had confirmed the presence of recurrent disease, no re-biopsy of the lymph nodes was performed. The patient was commenced on ibrutinib therapy (140 mg/day), and the swollen preauricular and cervical lymph nodes shrank rapidly. In addition, a CT scan demonstrated a significant reduction in the size of the right atrial mass within 4 months (Fig. 6). The patient continues to exhibit a partial remission and is currently receiving ibrutinib monotherapy.

## Discussion

The present study described the case of a patient with MCL; the patient presented with metachronous extranodal recurrences, involving the conjunctiva, soft tissue and right atrium. The conjunctival and soft-tissue lesions were pathologically evaluated by re-biopsies, and radiotherapy was beneficial. The most recent recurrence, involving the right atrium, was successfully treated with a tyrosine kinase inhibitor.

Ocular adnexal lymphoma is considered to be relatively rare, accounting for ~3% of extranodal non-Hodgkin lymphomas. On the other hand, lymphoma is one of the most common malignancies in the ocular adnexa, accounting for up to 55% of all primary malignancies in the orbit (6). As for the pathological subtype, according to the largest published study of ocular adnexal lymphoma (involving 353 cases), MCL is rare and comprises only 5% of ocular adnexal lymphomas (7). In these cases, secondary MCL affected the ocular adnexal region more commonly (63%) than primary (37%) MCL (7). As regards the therapeutic strategy, localized ocular adnexal lymphoma is generally treated with external-beam radiotherapy, while systemic disease is treated with chemoimmunotherapy alone or combined with radiotherapy. Radiotherapy is the standard treatment for isolated conjunctival lymphoma and this results in 5-year local control rates >80%. Although the optimal radiotherapy dose is unclear, doses >35 Gy exacerbate post-treatment toxicity and morbidity, while low, fractionated doses alleviate them (8). Concerning the survival rate, a previous study suggested that the 5-year disease-specific survival rates of patients with primary ocular adnexal MCL, systemic MCL with involvement of the ocular adnexa and relapsed systemic MCL in the ocular adnexal region did not differ (5-year disease-specific survival rate, 38%) (9). Although the disease recurred at different extranodal sites from the previous lesion in the case presented herein, administering radiotherapy at a dose of 40 Gy resulted in long-term local control since the disease only recurred in the conjunctiva.

As regards soft-tissue lymphoma, it is characterized by the disease growing within the soft tissue, which includes the connective tissue, adipose tissue and skeletal muscle. In a previous study, it reported to account for ~0.1% of all malignant lymphomas, although that study was limited to primary soft-tissue lymphomas (10). In a recent study, Trutzer and Lossos (11) described 4 cases of relapsed MCL affecting the soft tissue of the extremities. These 4 cases represented 0.85% of the MCL cases treated at their institution, highlighting the rarity of such cases (11). As regards diagnosis, several studies have suggested the usefulness of MRI for diagnosing soft-tissue lymphoma, e.g., lymphomas were found to be uniformly enhanced, exhibiting high signal intensity on T2-weighted imaging and intermediate intensity on T1-weighted images (12,13). On the other hand, a previous review that included both the abovementioned case series and previously reported cases described inconsistent MRI results (13). In the case in the present study, the internal MRI signals of the left popliteal fossa tumor were homogenous, and the tumor demonstrated intermediate signal intensity on T1-weighted images; however, the fact that the signal intensity of the tumor was not high on T2-weighted images was atypical. Therefore, it was considered that pathological diagnosis based on re-biopsy is preferable for improving the accuracy of diagnosis.

As for cardiac lymphoma, a single-center analysis of cardiac lymphoma was recently reported (14). Lymphomatous involvement in the heart was only found in 6 patients (1.5%; 5 patients with diffuse large B-cell lymphoma and 1 patient with B-cell lymphoma) among 394 patients who underwent echocardiography prior to chemotherapy (14). However, transthoracic echocardiography only has 60% sensitivity for detecting cardiac involvement (15), and in the case present herein, the cardiac mass could not be detected using echocardiography; hence, it was hypothesized that the actual incidence of the disease may be higher than reported. Concerning pathological diagnosis, it has been reported that diffuse large B-cell lymphoma, T-cell lymphoma and Burkitt lymphoma are the most common pathological diagnoses, in that order (16). There have been several reported cases of cardiac MCL similar to the present case report; Futela *et al* (17), who cited data from Surveillance, Epidemiology and End Results, mentioned the rarity of cardiac MCL. Anatomically, it has been reported that the right atrium, which was also affected in the case presented herein, is the most common location for cardiac MCL (15,18). In addition, Kudo *et al* (18) demonstrated that surgery is desirable to avoid the risk of a pulmonary embolism caused by the mass since cardiac lymphoma most frequently involves the right atrium. In the case in the present study, a CT scan only demonstrated the thickening of the right atrial wall; therefore, the continuation of ibrutinib treatment was possible without the concern of causing the mass to become dislodged as it shrank, resulting in rapid, safe and successful treatment. It is suggested that a therapeutic strategy including ibrutinib should be considered for the treatment of patients with cardiac MCL, particularly elderly patients and/or patients with a poor performance status, for whom surgery is not indicated.

Finally, as regards pathological evaluations, the positivity rate of Ki-67 immunohistochemistry is an independent prognostic factor distinct from the four MIPI factors, and a prognostic index that includes the Ki-67 positivity rate has also been proposed (5). In addition, aggressive MCL is characterized by rapid progression, frequent extranodal disease and a high Ki-67 positivity rate (19). In the case presented herein, although the Ki-67 positivity rate did not increase over time, which would suggest clonal evolution, Ki-67 positivity had been consistently observed since 2018, when a pathological evaluation became possible. The main limitation of the present case report was that it was not possible to demonstrate the possibility of clonal evolution through metachronous recurrence using FISH or Ki-67 positivity. However, it is considered that careful surveillance, including for the possibility of extranodal recurrence, is necessary when Ki-67 positivity is present, as demonstrated in the present case report.

## Data Availability

The data generated in the present study may be requested from the corresponding author.

## References

[1] Klapper W, Ferry JA, Hermine O, Li S, Lossos IS, Medeiros LJ, Naresh KN, Rosenquist R, Rule S, Stilgenbauer S

[2] Siebert R, Aukema SM

[3] Muscelli S, Shah SS, Bigcas JL (2023). Extranodal manifestation of mantle cell lymphoma in the submandibular duct: A case report and review of literature. Otolaryngol Case Rep.

[4] Ding YZ, Tang DQ, Zhao XJ (2022). Mantle cell lymphoma with endobronchial involvement: A case report. World J Clin Cases.

[5] Hoster E, Dreyling M, Klapper W, Gisselbrecht C, van Hoof A, Kluin-Nelemans HC, Pfreundschuh M, Reiser M, Metzner B, Einsele H (2008). A new prognostic index (MIPI) for patients with advanced-stage mantle cell lymphoma. Blood.

[6] Kirkegaard MK (2022). Ocular adnexal lymphoma: Subtype-specific clinical and genetic features. Acta Ophthalmol.

[7] Ferry JA, Fung CY, Zukerberg L, Lucarelli MJ, Hasserjian RP, Preffer FI, Harris NL (2007). Lymphoma of the ocular adnexa: A study of 353 cases. Am J Surg Pathol.

[8] Tanenbaum RE, Galor A, Dubovy SR, Karp CL (2019). Classification, diagnosis, and management of conjunctival lymphoma. Eye Vis (Lond).

[9] Knudsen MKH, Rasmussen PK, Coupland SE, Esmaeli B, Finger PT, Graue GF, Grossniklaus HE, Khong JJ, McKelvie PA, Mulay K (2017). Clinicopathological features of ocular adnexal mantle-cell lymphoma in an international multicenter cohort. JAMA Ophthalmol.

[10] Travis WD, Banks PM, Reiman HM (1987). Primary extranodal soft tissue lymphoma of the extremities. Am J Surg Pathol.

[11] Trutzer IM, Lossos IS (2024). Relapsed mantle cell lymphoma manifesting with soft tissue tumors of the extremities: University of Miami experience and review of the literature. Ann Hematol.

[12] Chun CW, Jee WH, Park HJ, Kim YJ, Park JM, Lee SH, Park SH (2010). MRI features of skeletal muscle lymphoma. AJR Am J Roentgenol.

[13] Spinnato P, Chiesa AM, Ledoux P, Kind M, Bianchi G, Tuzzato G, Righi A, Crombé A (2023). Primary soft-tissue lymphomas: MRI features help discriminate from other soft-tissue tumors. Acad Radiol.

[14] Ebina T, Sano Y, Hirabayashi M, Tsurumi T, Watanabe M, Furukawa M, Matsuo W, Nagasawa H, Hirose H, Horii M (2024). Echocardiographic findings of malignant lymphoma with cardiac involvement: A single-center retrospective observational study. Intern Med.

[15] Ikeda H, Nakamura S, Nishimaki H, Masuda K, Takeo T, Kasai K, Ohashi T, Sakamoto N, Wakida Y, Itoh G (2004). Primary lymphoma of the heart: Case report and literature review. Pathol Int.

[16] Gordon MJ, Danilova O, Spurgeon S, Danilov AV (2016). Cardiac non-Hodgkin's lymphoma: Clinical characteristics and trends in survival. Eur J Haematol.

[17] Futela P, Shabtaie SA, Woelber TJ, Poddar A, Deshmukh AJ, Kowlgi GN (2024). Mantle cell lymphoma with cardiac involvement presenting as complete heart block. JACC Case Rep.

[18] Kudo H, Shiroshita K, Shiozawa Y, Fujita S, Sakamoto M, Nakamura N, Nakanishi K, Toyama T (2024). Autopsy case of cardiac mantle cell lymphoma presenting with recurrent pulmonary tumor embolism after chemotherapy. J Clin Exp Hematop.

[19] Wilson MR, Barrett A, Cheah CY, Eyre TA (2023). How I manage mantle cell lymphoma: Indolent versus aggressive disease. Br J Haematol.

